# Insight into Desorption Mechanisms in a Helium Low-Temperature Plasma Ionization Source Using Computational Simulations

**DOI:** 10.1021/jasms.5c00171

**Published:** 2025-09-12

**Authors:** Odhisea Gazeli, Constantinos Lazarou, Marcos Bouza, David Moreno-González, Charalambos Anastassiou, Joachim Franzke, Juan F. García-Reyes, George E. Georghiou

**Affiliations:** † FOSS Research Centre for Sustainable Energy, Department of Electrical and Computer Engineering, 54557 University of Cyprus, Nicosia 1678, Cyprus; ‡ ENAL Electromagnetics and Novel Applications Lab, Department of Electrical and Computer Engineering, University of Cyprus, Nicosia 1678, Cyprus; § Analytical Chemistry Research Group, Department of Physical and Analytical Chemistry, 16747 University of Jaén, 23071 Jaén, Spain; ⊥ 28371 Leibniz-Institut für Analytische Wissenschaften − ISAS − e.V., Bunsen-Kirchhoff-Str. 11, 44139 Dortmund, Germany

**Keywords:** Ambient Ionization Mass Spectrometry, Low-Temperature Plasma (LTP), Computational Simulation, Desorption Mechanisms, Dielectric Barrier Discharge (DBD), Plasma−Surface Interaction

## Abstract

Understanding desorption mechanisms is essential for the optimization of analytical techniques that enable the direct sampling and ionization of condensed-phase samples without preparation. The low-temperature plasma (LTP) ionization source, first described by Harper (Harper; et al. Anal. Chem. 2008, 80, 9097–9104 19551980 10.1021/ac801641a) and based on the dielectric barrier discharge principle, is among the more representative and replicated plasma-based ambient desorption/ionization tools for mass spectrometry (MS), although there are a wide array of designs and configurations. However, the fundamental desorption mechanisms directly associated with LTP and other related plasma-based sources remain unclear. In this study, we utilized plasma simulations using COMSOL Multiphysics to understand analyte release from solid samples placed on glass and exposed to the plasma of a simplified helium LTP configuration. Our simulations revealed that the accumulation of surface charge on the sample–substrate that is caused by the plasma results in localized electric fields strong enough to likely aid in disruption of analyte–substrate interactions and facilitate desorption. Importantly, our model estimates that electrons in plasma have energies of approximately 2.5 eV, suggesting this simulated energy level is an indicator for desorption efficiency. Our findings provide new insight into the complex interplay between plasma-induced phenomena and desorption processes.

## Graphical abstract

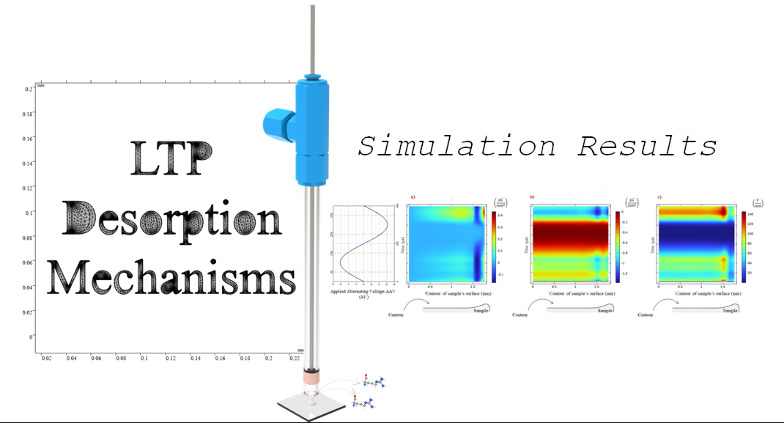

## Introduction

Ambient desorption ionization mass spectrometry (ADI-MS) is an approach that allows rapid MS analysis of samples in their natural state without requiring detailed sample preparation and, usually, with no significant damage.^1^ Unlike traditional desorption/ionization (D/I) MS methods, which occur under vacuum in an enclosed environment, ADI-MS can be performed under ambient conditions, allowing for direct analysis of solid, liquid, and gaseous samples.^1–3^ ADI-MS is applied using a variety of desorption methods, the most widely used being charged droplet-based,^4^ plasma-based,^5^ and laser-based desorption.^6^

Plasma-based D/I sources are one of the more relevant classes of ADI-MS.^5^ Out of these, low-temperature plasma (LTP) stands out as particularly significant.^7,8^ LTP works as a current-driven plasma distinguished by its nonthermal or cold plasma characteristics. This property arises from the notably lower temperatures produced by the LTP compared to thermal plasmas, which typically reach around 10,000 K.^9^ Beyond its uses for ADI-MS,^8,10^ the LTP, based on a dielectric barrier discharge assembly, has a wide range of applications due to its unique characteristics, including surface treatment and sterilization.^11–13^

LTP is commonly employed in ADI-MS due to the ability to generate ions, radicals, and excited atoms. This makes them suitable for analyzing a broad spectrum of compounds including polar and nonpolar and low to medium molecular weight molecules (e.g., 50–500 Da).^14,15^ Additionally, LTP is ideal for examining temperature-sensitive samples, such as biomedical materials.^16^ A typical low-temperature plasma ionization source setup consists of a dielectric glass, commonly cylindrical in shape, along with two electrodes: one operating at high alternating voltage (AV) with variable frequency and another grounded. This setup requires a working gas, typically helium. The reagent ions produced by the LTP plasma result from the interaction between the electric field and the working gas. The strong electric field induces the ionization of the working gas, giving rise to radical species and positive and negative ions. One of the appealing aspects of the LTP probes is their cost-effective construction and minimal power and reagent consumption. They can also be adapted for use in hand-held and portable systems.^17^

The mechanisms of desorption and ionization in LTP are still not fully understood and constitute an area of research.^14,15,18^ However, publications have evaluated the analytical performance of LTP,^10,18^ even for challenging analytes. Explosives, characterized by low vapor pressures and high boiling points, were directly detected using either the first LTP probe^19^ or related DBDI-based configurations.^20^ Compounds such as pentaerythritol tetranitrate, trinitrotoluene (TNT), tetryl, and cyclo-1,3,5,7-tetramethylenetetranitrate (HMX) were detected at low levels (ranging from 0.6 pg for TNT to 0.6 ng for HMX). Desorption mechanisms were proposed to occur through the sputtering of surfaces by reactive plasma species and the interaction of helium species with the samples, leading to the release of analytes from the surfaces.^18^ Furthermore, Chan et al.^21^ conducted optical emission spectroscopy experiments to investigate D/I mechanisms responsible for the performance of LTP. This study aimed to explore the pathways for the production of N_2_^+^ ions in the LTP probe. The authors proposed a set of reaction mechanisms for afterglow formation, including charge transfer between N_2_^+^ ions, which form the N_2_^+^ molecular ion. They found that Penning ionization from metastable helium is not the sole ionization pathway for N_2_^+^ ion formation and that He_2_^+^ ions act as carriers to transfer energy from the plasma to the afterglow region, where the sample is positioned.

The mechanisms governing analyte desorption in plasma-based ambient ionization sources are recognized as being complex and multifactorial, generally resulting from a combination of processes including thermal effects, momentum transfer from the gas stream, and the impact of energetic plasma species on the surface.^22^ Specifically for LTP, suggested mechanisms include He metastable energy-transfer, ion impact, and radical–surface interactions.^18^ However, as highlighted in a seminal review by Chen et al., definitive experimental evidence for these processes remains limited, and “a more comprehensive study is also needed for showing more supporting evidence for ionization and desorption mechanisms”.^22^ Notably, the same review observed that varying the interelectrode distance in an LTP source (thereby altering the accelerating electric field) directly influenced analyte fragmentation, pointing toward the critical and under-investigated role of electric field interactions.

This study directly addresses this need for a deeper investigation by presenting a numerical model that provides insight into the complex interplay between plasma-induced phenomena and desorption processes. We simulate the flow dynamics and plasma chemistry within a simplified but representative LTP configuration, which is closely aligned with the electrical properties of the probe described by Harper et al.^7^ Our model captures the qualitative spatiotemporal distributions of various species and, crucially, the resulting electric field distribution within the LTP assembly and the displacement field within the sample. To manage numerical instabilities and ensure computational feasibility, we made certain adjustments to the probe geometry, as detailed in subsequent sections. While these adaptations may introduce minor deviations from the original design, they do not compromise the fundamental insights of the model and, in fact, enhance its applicability to a broader class of similar helium LTP devices.^23,24^ By maintaining a focus on the underlying physics and their impact on experimental observations, this study contributes to a more fundamental understanding of LTP and its potential applications in MS.

## Experimental Section

### Description of the Simulation Domain

The numerical model used in this study simulates the LTP like in previous simulations described elsewhere.^25,26^ However, modifications were made to represent, as close as possible, the geometry and behavior of the LTP probe for surface desorption applications. The model configuration was based on the physical design of the LTP probe, which reported experimental measurements of samples placed on a glass substrate in front of an MS inlet under ambient conditions.^7,10^ COMSOL Multiphysics software was employed in a 2D-Axisymmetric configuration to simulate the probe’s plasma discharge and interaction with the sample surface, allowing investigation of desorption mechanisms.

The LTP probe consists of a borosilicate capillary glass and two electrodes. The glass capillary had an outer diameter of 6.35 mm and an inner diameter of 3.75 mm. In the simulation, the capillary is modeled as a rectangle positioned off the axis with a relative permittivity (ε_r_) of 6.7, which is a typical value for borosilicate glass.^27^ The first electrode surrounds the outer surface of the glass capillary while it operates at an applied alternating voltage of 6.2 kV and frequency of 2.5 kHz (we will refer to the applied alternating voltage as AAV onward). This electrode is represented by a 1 mm long line tangential to the outer edge of the glass capillary rectangle. The second electrode is positioned inside the capillary along the axis of symmetry. It has a pin shape with a diameter of 1.57 mm and serves as the ground electrode. This inner electrode is modeled as a curved line. Figure 1 illustrates the numerical domain of the LTP probe used in this simulation. The glass capillary rectangle is shown in cream-colored region, and the two electrode representations are depicted as black lines (the outer electrode as a black line positioned between numbers 8 and 9, and the inner electrode as a black line positioned between numbers 1 and 2). This simplified model enables for the simulation of the electric field interaction between the electrodes through the glass capillary.

**Figure 1:**
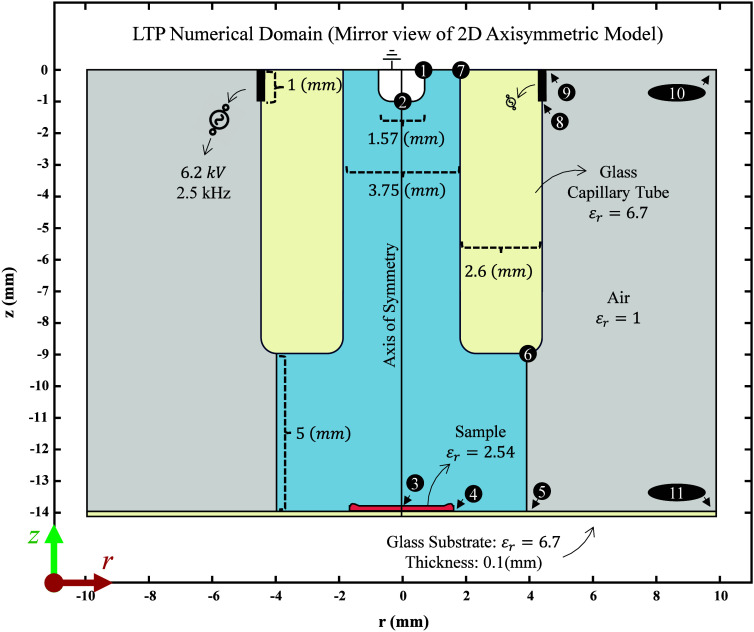
2D-Axisymmetric representation of the simulation domain used to model the LTP probe desorption mechanisms (the positive *z*-direction points upward, and the positive *r*-direction points radially outward). The light blue area represents the region where the plasma is created and propagated surrounded by ambient air (depicted in light gray). The dielectric glass capillary tube is shown in cream, and the sample (in orange) is placed on the glass substrate. The central vertical line indicates the Axis of Symmetry. Key physical and electrical parameters are displayed; the sample has a dielectric constant (ε_r_) of 2.54, and both the glass substrate and the capillary tube have a dielectric constant of 6.7. The distance between the probe and the substrate is 5 mm, and an applied alternating voltage (AAV) of 6.2 kV at a frequency of 2.5 kHz is used. The numbers in black circles correspond to the boundaries, whose conditions are defined in Table 1.

To accurately model the sample of analysis, a disk-shaped geometry was created based on the coffee ring effect. This effect refers to the deposition of particulate matter in a ring-like pattern that occurs when droplets of liquid containing solutes dry on a surface. To replicate this characteristic ring shape in the simulation, a disk geometry was used with varying thickness, tapering thinner in the central region and thicker around the edges (see Figure 1 yellow area between numbers 3 and 4). This disk was positioned coaxially atop a 0.1 mm thick layer representing the underlying borosilicate glass substrate (see Figure 1 cream region area under the sample). By numerically reproducing the coffee ring profile left after droplet drying, the sample geometry incorporated a realistic deposition pattern.

It is important to clarify that in this simulation we do not treat the sample as individual molecules capable of interacting with each other. Instead, we modeled the sample as a solid volume of pure analyte, which after solvent evaporation appears like a solid crystal to the naked eye. The simulation aimed to investigate the polarization of the sample, i.e., the macroscopic behavior of molecules when exposed to plasma, rather than focusing on their microscopic interactions. Therefore, the modeling is conducted using the dielectric constant (ε_r_) of 2.54 as reference. This is similar to the predicted bulk dielectric constant of l-arginine crystals, as previously calculated by Guerin.^28^

The numerical model of the LTP probe is based on its geometrical characteristics and consists of two different simulations. The first simulation models the gas flow dynamics inside and outside of the probe (see the light blue and light gray regions in Figure 1). It calculates the velocity profile and mass fractions of helium and air mixtures as they emerge from the probe. The outputs of this gas flow simulation serve as initial conditions for the second simulation of the model: the plasma/discharge simulation.

This simulation is computationally expensive and is solved in a reduced region of space that is represented by the light blue color in Figure 1. Figure 2 provides a visual representation of the two coupled parts of the model, highlighting the inputs and outputs of each part.

**Figure 2:**
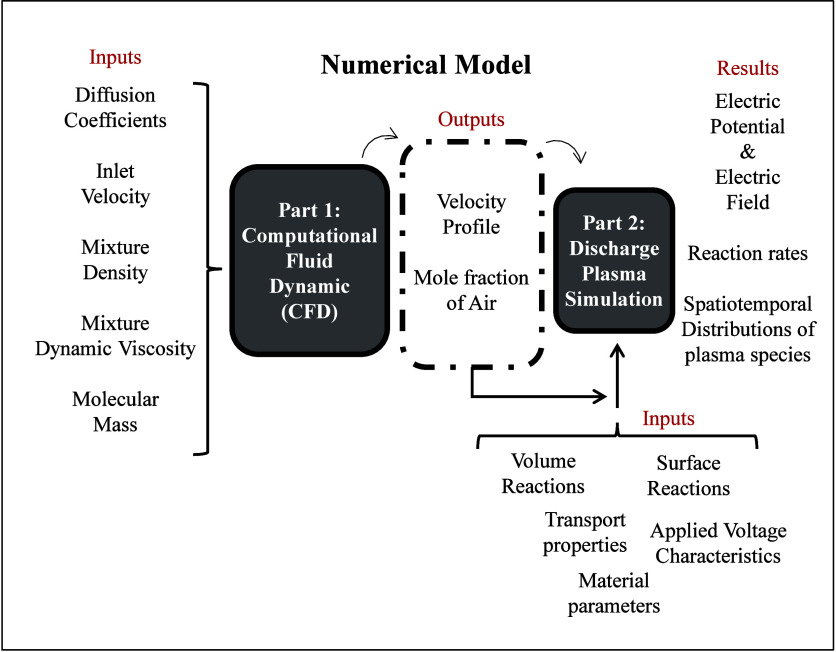
Schematic representation illustrating the coupling of two simulations in the numerical model. The output of the plasma/discharge model provides information about the electrical and chemical characteristics of the LTP probe.

### Description of the Numerical Model. Part 1: Gas Dynamic Model

For this model, the continuity equations of the mass and momentum for the He-Air mixture are solved in steady-state formulation:

1 ∇·(ρu)=0

2 $$\rho \left(u\cdot \nabla \right)u=\nabla \cdot \left[-\nabla PI+\mu \left(\nabla u+\nabla u^{T}\right)-\frac{2}{3}\mu \left(\nabla \cdot u\right)I\right]$$

where ***u*** represents velocity, *P* is the pressure, ***I*** is the identity matrix, and μ stands for the dynamic viscosity of the He-Air mixture.^29^ The mixture’s density is expressed as ρ = Σ_*i*_ *n*_*i*_ *m*_*i*_, where *m*_*i*_ is the molecular mass of the *i* ^th^ species and *n*_*i*_ is the number density of the *i* ^th^ species. The mass fractions ω_*i*_ for species *i* are given by the conservation of mass equation:

3 $$\rho \left(u\cdot \nabla \right)\omega_{i}=\nabla \cdot \left(\rho \frac{m_{i}}{\underset{i}{\sum }m_{i}x_{i}}D_{mi}\nabla x_{i}\right)$$

where *x*_*i*_ is the mole fraction of the *i* ^th^ species and *D*_*mi*_ is the effective diffusion coefficient of the *i* ^th^ species with the mixture, defined by:^26^

4 $$D_{mi}=\frac{1-\omega_{i}}{\underset{k\neq i}{\sum }\frac{x_{k}}{D_{ik}}}$$

where *D*_*ik*_ is the binary diffusion coefficient for the *i* ^th^ species in *k* ^th^ species, determined from the kinetic theory of gases.^30^ The value of the binary diffusion coefficients used in the flow dynamics model was $7.44\cdot 10^{-5}\left(\frac{m^{2}}{s}\right)$ . The flow dynamics model is solved in the hole domain shown in Figure 1 (the entire computational domain), using the boundary conditions for the flow dynamics model collected in Table 1.

**Table 1:** Boundary Conditions for the gas dynamic Model

Boundary	Flow Condition	Species Condition
1–7	Inlet	Fixed mass fraction
	$u_{r}=0,u_{z}=0.6\;\left(\frac{m}{s}\right)$	He = 1
1–2	Wall	No flux
	*u*_*r*_ = 0, *u*_*z*_ = 0	***n*** ***Γ***_***i***_ = 0
2–3	Symmetry	Symmetry
	$\frac{\partial u_{r}}{\partial r}=\frac{\partial u_{z}}{\partial r}=\frac{\partial P}{\partial r}=0$	$\frac{\partial \omega_{i}}{\partial r}=0$
3–4	Wall	No flux
	*u*_*r*_ = 0, *u*_*z*_ = 0	***n*** ·Γ_***i***_ = 0
4–5–11	Wall	No flux
	*u*_*r*_ = 0, *u*_*z*_ = 0	***n*** · ***Γ***_*i*_ = 0
6–8–9	Wall	No flux
	*u*_*r*_ = 0, *u*_*z*_ = 0	***n*** · ***Γ***_*i*_ = 0
8–9–10	Constant pressure, zero viscus stress	
	*P* = 1 atm	Fixed mass fraction
	$\left[\mu \left(\nabla u+\nabla u^{T}\right)-\frac{2}{3}\mu \left(\nabla \cdot u\right)I\right]\cdot \mathrm{n̂}=0$	Air = 1

To save computational time and because the diffusion coefficients are not so easy to find in the literature, we choose to not add the impurities of the working gas (Helium in our case) in this model. The impurities are considered in the plasma fluid model using the mass fraction of air calculated from the output of this model. This approach simplifies the building of the model and saves time from the final calculation of the results.

### Description of the Numerical Model. Part 2: Plasma Fluid Model

In the discharge model, the electron density (*n*_*e*_), the species densities (*n*_*i*_), the average electron energy density (*n*_ε_), and the electric potential of the plasma (*V*) are calculated in the region marked in light blue in Figure 1. The surface charge density (ρ_*s*_) is determined by integrating the electron flux (*Γ*_*e*_) and the total flux (*Γ*_*i*_) of ions reaching various surfaces over time. This study applies a sinusoidal AAV with an amplitude of 6.2 kV and a frequency of 2.5 kHz to the anode.

The plasma model calculates the densities for 15 species through 113 reactions (see Supporting Information), including e, He, He*, He^+^, He_2_, He_2_^+^, N_2_, N_2_^+^, O_2_, O^–^, O_4_^+^, O_2_^–^, O_2_^+^, N_4_^+^, He­(2P^3^P). Although other species could be included in practice, this study focuses on critical reactions and the most important species for computational efficiency. Details of these reactions, their rates, and energies are provided in Table S-1 (Supporting Information), along with the surface reactions caused on the dielectric surfaces in Table S-2 (Supporting Information). The initial electron density for this model was uniform, and the value was $10^{12}\left(\frac{1}{m^{3}}\right)$ .

The plasma model incorporates the continuity equation in the drift diffusion approximation to account for electrons and their energy:

5 $$\frac{\partial n_{e}}{\partial t}+\nabla \cdot \Gamma_{e}=S_{e}-\left(u\cdot \nabla \right)n_{e}$$

6 $$\frac{\partial n_{\varepsilon }}{\partial t}+\nabla \cdot \Gamma_{\varepsilon }=-e\Gamma_{e}\cdot E+S_{\varepsilon }-\left(u\cdot \nabla \right)n_{e}$$

where *Γ*_ε_ represents the flux of electron energy, *E* is the electric field, *S*_*e*_ is the source term for the electron production/loss, *S*_ε_ is the source term that accounts for the energy gain or loss in elastic and inelastic collisions of electrons with the heavy species in the mixture, and *u* is the mass average velocity of the mixture. The mass fraction of heavy species in the mixture is determined by the multicomponent diffusion equation:

7 $$\rho \frac{\partial }{\partial t}\left(\omega_{i}\right)+\rho \left(u\cdot \nabla \right)\omega_{i}=\nabla \cdot j_{i}+S_{i},i=1,2,...,Q-1$$

where ρ is the density of the mixture, ω_*i*_ is the mass fraction of species *i*, *j*_*i*_ is the diffusive flux vector, *S*_*i*_ is the source term, and *Q* is the number of heavy species in the mixture. The density of the background gas (helium 99.996% purity) is calculated from:

8 $$\omega =1-\overset{Q-1}{\underset{i=1}{\sum }}\omega_{i}$$

while the impurities (40 ppm) of the air from the bottle are calculated based on the following relationships:

9 $${\left(N_{2}\right)}_{impurities}=\left({\left(\omega_{air}\right)}_{GDMO}+\left(1-{\left(\omega_{air}\right)}_{GDMO}\right)\times 0.00004\right)\times 0.79$$

10 $${\left(O_{2}\right)}_{impurities}=({\left(\omega_{air}\right)}_{GDMO}+\left(1-{\left(\omega_{air}\right)}_{GDMO}\right)\times 0.00004)\times 0.21$$

where the index GDMO indicates that the mass fraction ω_air_ is the output of the gas dynamic model that is described in the previous section. The electric field in the volume of plasma is calculated from Poisson’s equation:

11 $$-\nabla D=\rho_{v}$$

where ***D*** represents the electric displacement field and ρ_*v*_ is the volume charge density.

The boundary conditions for the discharge/plasma simulation are presented in Table 2.

**Table 2:** Boundary Conditions for the Plasma Model

Boundary	Electrostatic Condition	Species Condition	Electrons Condition
1–7	Field continuity	Continuity	Continuity
	***n*** ***E*** = 0	***n*** · ***Γ*** = 0	***n*** · ***Γ*** = 0
1–2	Constant potential	Wall losses	Wall losses^a^
	*V* = 0	$n\cdot \Gamma_{i}=\frac{\gamma_{i}}{4-2\gamma_{i}}n_{i}v_{th,i}+\rho \omega_{i}\mu_{i}{Ε}_{n}$	$n\cdot \Gamma_{e}=\frac{1}{2}n_{e}v_{e,th}-\underset{i}{\sum }\gamma_{i}\left(\Gamma_{i}\cdot n\right)$
			$$n\cdot \Gamma_{\varepsilon }=\frac{5}{6}n_{e}v_{e,th}-\underset{i}{\sum }\gamma_{i}\varepsilon_{i}\left(\Gamma_{i}\cdot n\right)$$
2–3	Symmetry	Symmetry	Symmetry
	*E*_*r*_ = 0	$\frac{\partial n_{i}}{\partial r}=0$	$\frac{\partial n_{e}}{\partial r}=\frac{\partial n_{\varepsilon }}{\partial r}=0$
3–4–5–6–7	Field continuity	Wall losses	Wall losses
	***n*** ·(ε_1_ ***Ε***_**1**_ – ε_2_ ***Ε***_**2**_) = ρ_*s*_	$n\cdot \Gamma_{i}=\frac{\gamma_{i}}{4-2\gamma_{i}}n_{i}v_{th,i}+\rho \omega_{i}\mu_{i}{Ε}_{n}$	$n\cdot \Gamma_{e}=\frac{1}{2}n_{e}v_{e,th}-\underset{i}{\sum }\gamma_{i}\left(\Gamma_{i}\cdot n\right)$
			$$n\cdot \Gamma_{\varepsilon }=\frac{5}{6}n_{e}v_{e,th}-\underset{i}{\sum }\gamma_{i}\varepsilon_{i}\left(\Gamma_{i}\cdot n\right)$$
6–8	Field Continuity		
	***n*** ·(ε_1_ ***Ε***_**1**_ – ε_2_ ***Ε***_**2**_) = 0		
8–9	Constant Potential		
	*V* = *V*_*rf*_ (6.2 kV, 2.5 kHz)		
9–10–11	Field continuity		
	***n*** ***E*** = 0		

a γ_*i*_: secondary emission coefficient; *v*_*th*_: thermal velocity; μ_*i*_: mobility; ε_*i*_: mean energy.

### Description of the Spatiotemporal Discretization

To solve the described models, a mesh was utilized with the following characteristics: a triangular mesh size of 0.05 mm was used in areas where the continuity and drift-diffusion equations were solved, while a larger triangular mesh size of 2.28 mm was used in areas where the volume charge density was expected to be low. The mesh size was limited to 0.01 mm in areas characterized by boundary conditions 2–3 and 6–7 (Figure 1) and to 0.0075 mm in areas characterized by boundary conditions 1–2 and 3–4–5 (Figure 1). As a result, the mesh contained 183,619 triangular elements, corresponding to 1,508,243 degrees of freedom (DoF).

The characteristics of the aforementioned mesh illustrate why it was not feasible to model the standard dimensions of the LTP probe.^10^ Specifically, modeling the probe’s realistic centimeter-scale geometry would require resolving plasma phenomena, such as ionization waves, that occur over scales of tens of micrometers. This necessitates an exceptionally fine mesh. For instance, capturing the evolution of the ionization wave along the 20 mm axis of symmetry alone would require at least 2000 nodes. Extending this mesh density across the entire 2D axisymmetric computational domain, which has dimensions of several centimeters, would result in an exponential increase in the number of mesh elements. Given that multiple plasma equations are solved at each node (one for each species of plasma), this would cause the total degrees of freedom to become computationally prohibitive, vastly exceeding the already substantial 1.5 million DoF of our simplified model. Furthermore, using a coarser mesh is not a viable alternative, as it leads to severe numerical instabilities in the solver.^35,36^ For these compelling reasons, reducing the interelectrode distance was a necessary compromise to ensure both computational feasibility and numerical stability.

Regarding temporal discretization, the time interval of interest, that is, one period of AAV, from 0 to 400 μs, was divided into 2,000 equal time steps. The numerical problem was solved using a computer equipped with an octacore processor (Intel Xenon E5–2667 V4 3.2 kHz), 252.2 GB of memory, and 5.5 Tbyte of storage space.

The differential equations described above were discretized with the Galerkin method^31^ using linear shape functions. The time taken for the gas dynamic simulation of the model to solve was 30 min, while for the discharge/plasma simulation, it was 2 days, 12 h, and 49 min.

### Model Assumptions and Limitations

It is important to acknowledge that any numerical model is an approximation of a complex physical reality. The findings presented in this study are subject to certain assumptions and limitations inherent to the modeling approach, which are outlined below to provide a clear context for the interpretation of our results.

A primary limitation of our model is the necessary simplification of the LTP probe’s geometry, specifically the reduction of the distance between the internal and external electrodes. This adjustment was implemented to render the problem computationally achievable. The physics of atmospheric pressure plasma discharges, particularly the propagation of ionization waves, involves extremely high velocities and the formation of very thin space-charge layers with steep gradients in both species densities and electric field. To accurately resolve these phenomena, a numerical model requires an exceptionally fine mesh, with element sizes often less than 10 μm. When such a fine mesh is applied over a realistic, centimeter-scale plasma reactor, the total number of degrees of freedom can become computationally prohibitive. Furthermore, the tightly coupled, highly nonlinear nature of the governing plasma fluid equations can lead to numerical instabilities if the mesh is not sufficiently dense to resolve these sharp gradients.^35^ For these reasons, geometric reduction was a necessary compromise. While this simplification may affect the precise quantitative outputs, it preserves the fundamental physics. The model remains robust in capturing key qualitative trends and mechanisms, such as surface charge accumulation and the resulting electric field enhancement, which are the central focus of this investigation.

The conclusions drawn in this study are specifically applicable to the system modeled: helium-based, low-temperature DBDs of various frequencies and voltages interacting with a solid analyte on a glass substrate. Extrapolation of these findings to other ambient ionization systems must be performed with significant caution. For instance, sources such as Direct Analysis in Real Time (DART) operate in a fundamentally different regime, primarily driven by a high-temperature gas stream rich in long-lived helium metastables where thermal desorption and Penning ionization are the dominant mechanisms. The plasma-induced electric field effects detailed in our work likely play a secondary role. To the best of our knowledge, comparable, fully coupled fluid dynamics and plasma chemistry simulation for the DART desorption mechanism have not been reported, highlighting its distinct complexity.

Furthermore, plasma chemistry is critically dependent on the working gas. Changing the gas from helium to argon, for example, would require a complete reformulation of the chemical model, as an argon plasma jet involves a unique set of reactions and metastable states that lead to different discharge characteristics.^32^ Similarly, the inclusion of molecular gases such as nitrogen and oxygen (i.e., air) introduces vast and complex reaction networks. Our own previous work has demonstrated that even trace admixtures of air, oxygen, or water vapor in a helium discharge necessitates many additional reactions to accurately capture the plasma evolution, significantly altering the dominant ion species and discharge dynamics.^33–35^

Finally, the desorption mechanism we propose is intrinsically tied to the accumulation of charge on a dielectric surface. The use of a conductive surface, such as stainless steel, would prevent this charge buildup by allowing charges to dissipate rapidly, thereby inhibiting the formation of the localized electric fields that we identify as a key driver for desorption. Modeling interactions with complex substrates like biological tissues would present even greater challenges, requiring the consideration of factors such as high water content, intricate surface topography, and heterogeneous electrical properties, which are far beyond the scope of this study.

## Results and Discussion

### Gas Dynamic Model Results

The first part of the model focused on the dynamic behavior of the working gas, revealing key phenomena that influence the final plasma configuration. The simulation models a jet of pure helium flowing from the capillary exit into stationary ambient air. The resulting velocity profile and gas mixing are listed in Figure 3. A relatively high-velocity core jet, composed primarily of helium and reaching approximately 1 m/s, flows toward the sample surface (Figure 3a). A relatively high-velocity helium jet, reaching approximately 1 m/s, flows from the capillary exit toward the sample surface. On the periphery of the main jet, the interaction with the stationary ambient air creates recirculation zones (vortices), which facilitate gas mixing. Figure 3b,c illustrates the mass fractions of helium and air, respectively. These plots show that, while the core of the jet remains helium-rich, significant mixing with air occurs near the capillary outlet and in the recirculation zones, as highlighted by the “Mixing” annotations. It is important to note that the arrows in Figure 3a represent the velocity of the bulk gas mixture, which is dominated by outflowing helium, while air enters the stream primarily via diffusion and convection in these turbulent mixing regions.

**Figure 3:**
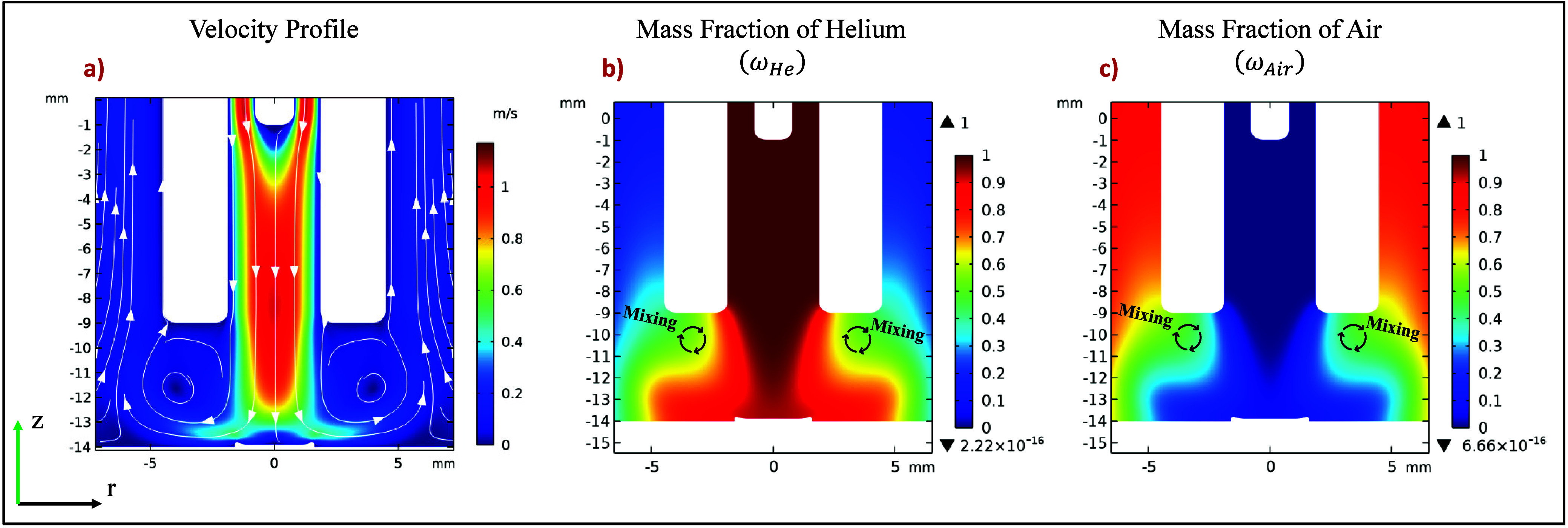
Visualization of the gas dynamic simulation results. (a) Spatial profile of the gas mixture velocity; white arrows indicate the bulk flow direction, while the color map represents the velocity magnitude (m/s). (b) Mass fraction of helium (ω_He_) and (c) mass fraction of air (ω_air_). The mass fraction is defined as the mass of a given species divided by the total mass of the mixture at any given point. The simulation models a jet of pure helium (100% He at the inlet) exiting into stationary ambient air. Mixing between the two occurs via diffusion and convection, particularly in the turbulent regions indicated by the circular arrows.

Apart from this behavior, we noticed a substantial decrease in helium speed as the discharge gas approaches the surface of the sample (Figure 3a). This stagnation of the flow in front of the surface leads to the formation of a region of locally higher static pressure, which in turn causes the gas to expand radially, forming two lobes that maintain a speed close to 0.5 m/s. This flow pattern is crucial as it dictates the final mixture composition of He and Air in the region where the plasma interacts with the sample.

### Calculation of Air Impurities for the Plasma Model

Based on the calculated mass fraction of air from the gas dynamic simulation (Figure 3c), the initial mass fractions of the primary air impurities, nitrogen (N_2_) and oxygen (O_2_), were determined using eqs 9 and 10. The resulting spatial distribution of these impurities, which serve as crucial inputs for the subsequent plasma fluid model, is shown in Figure 4. This two-step approach ensures that the plasma simulation accurately accounts for the presence of air that has been entrained into the helium stream, providing more realistic initial conditions for the discharge simulation.

**Figure 4:**
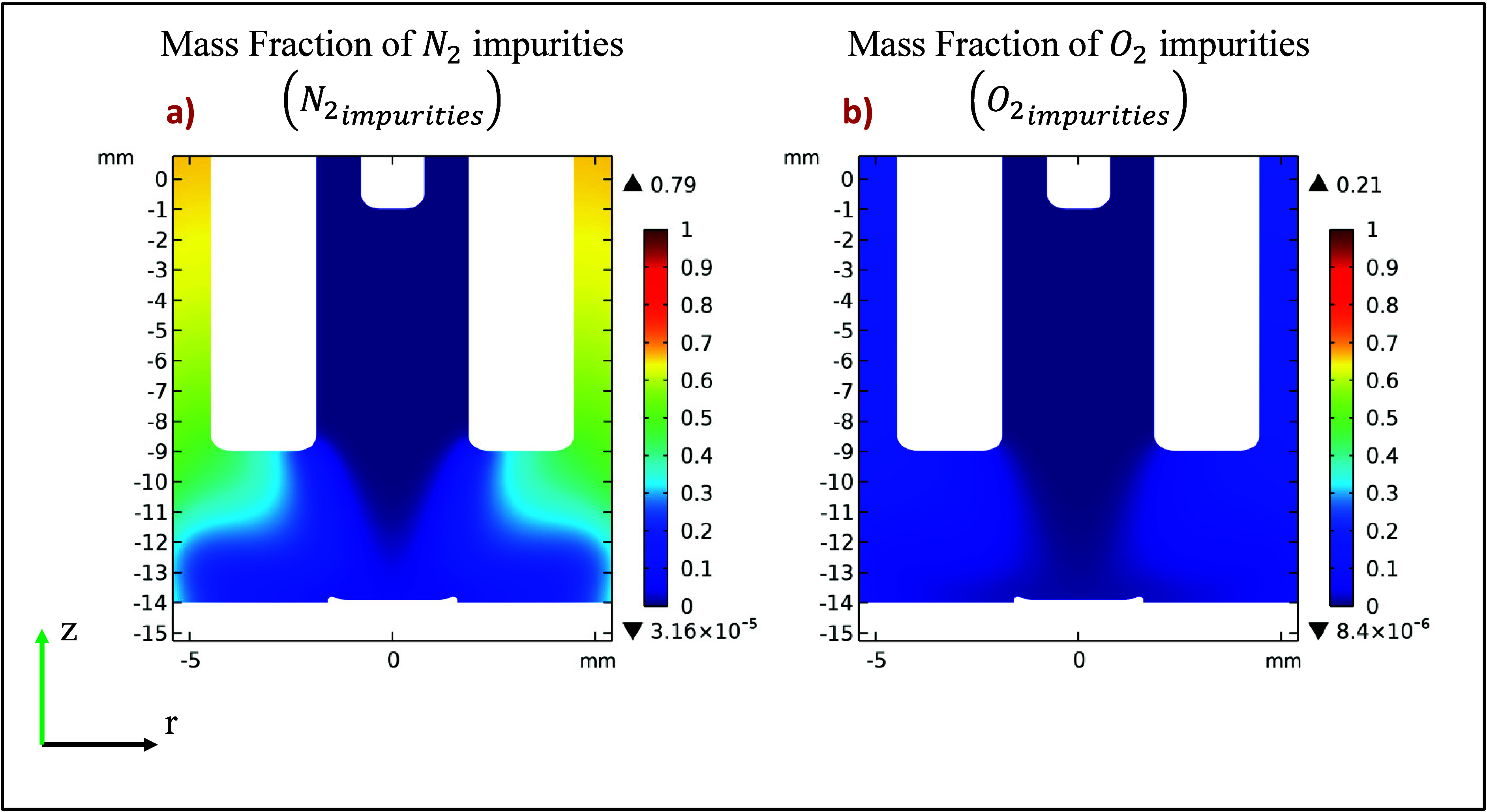
Spatial distribution of the mass fractions for (a) N_2_ and (b) O_2_ impurities, as calculated from the gas dynamic model results using eqs 9 and 10. These distributions were used as the initial conditions for the plasma fluid model.

### A Note on Data Visualization

The plasma fluid model calculates the spatiotemporal distribution of various species, whose number densities often span several orders of magnitude across the simulation domain. Visualizing such a wide dynamic range using a linear color scale would obscure important details, as regions with lower densities would be indistinguishable. To address this and provide a clearer representation of the plasma structure and dynamics, all subsequent contour plots illustrating species densities (e.g., electrons and ions) are presented using a logarithmic color scale. This approach effectively highlights subtle but significant variations across the entire plasma volume. Consequently, the values shown on the color bars of Figures 5 to 9 represent the natural logarithm of the number density (ln­(*n*)), where the density *n* is expressed in units of m^–3^. For the reader’s convenience, the exact correspondence between the logarithmic values shown on the color bars and the absolute number densities (in m^–3^) is provided in Table 3.

**Table 3:** Correspondence between the Logarithmic Values and Absolute Number Densities.

ln(*n*)	Number Density *n* (m^–3^)
26	2 × 10^11^
28	1.5 × 10^12^
30	1.1 × 10^13^
32	7.9 × 10^13^
34	5.8 × 10^14^
36	4.3 × 10^15^
38	3.2 × 10^16^
40	2.4 × 10^17^
42	1.7 × 10^18^
44	1.3 × 10^19^

**Figure 5:**
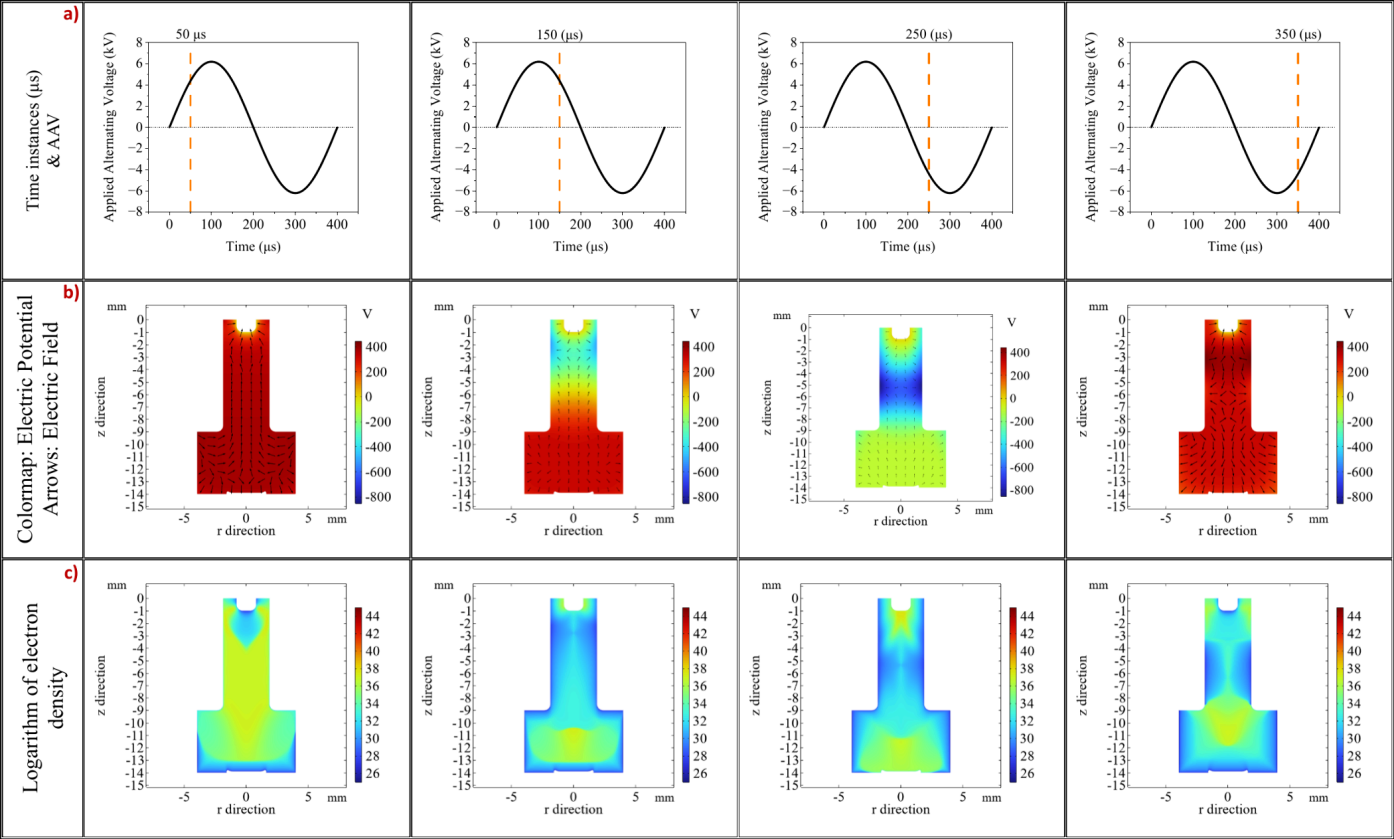
Evolution of the electric potential and electron density during an AAV cycle. (a) Four time instances of interest during the AAV cycle depicted by orange lines; from this point on, the four time instances (50, 150, 250 and 350 μs) were used to evaluate the changes of the different parameters and plasma species during the simulation, shown beneath each time instance. (b) Electric potential evolution at the same time instances of the AAV. (c) Electron density evolution for the complete AAV cycle. The color bar represents the natural logarithm of the number density (ln­(*n*_*e*_)). The displayed logarithmic values correspond to an absolute electron density range from approximately 2.0 × 10^11^ m^–3^ (ln­(*n*) ≈ 26) to 1.3 × 10^19^ m^–3^ (ln­(*n*) ≈ 44).

### Plasma Fluid Model Results. Spatiotemporal Distribution of Species

The simulations related to the LTP’s plasma examined the spatiotemporal characteristics of key plasma parameters such as electric potential, electric field, and species densities. Additionally, supplementary animations to visualize the dynamic evolution of plasma throughout the simulated discharge cycle were supplied. When these dynamic images are observed (Figures S1–S6, Supporting Information), we can provide a clearer understanding of how the plasma changes rapidly in both space and time for the LTP probe.

The distribution of the electric potential during a complete AAV cycle (Figure 5a) suggests the soft nature of the ionization. As shown in Figure 5b, the potential within the plasma volume is 1 order of magnitude lower than the AAV applied to operate the ion source. Specifically, near the sample area, the plasma potential approaches zero for the time interval between *t* = 150 and 250 μs of the AAV cycle. At 350 μs, the potential increases again to around 400 V. This indicates that ionization of the working gas is weak, keeping the plasma potential low throughout the AAV period.

During a cycle of the AAV, the electron density within the plasma volume changes dynamically (Figure 5c). Initially (*t* = 50 μs) when the AAV was positive, a strong production of electrons was observed throughout the volume of the LTP probe and in the exit of the capillary tube close to the sample. This surge is attributed to the inner electrode acting as a ground, attracting free electrons from the He-Air mixture and resulting in an increase of electron density within the volume where the working gas is present. As the AAV changes to negative values (*t* = 150 μs), the electron density inside the dielectric tube decreased by approximately 4 orders of magnitude, remaining constant near the sample surface. Moreover, at *t* = 250 μs, a strong interaction between the surface of the sample and the electrons of the plasma was observed, causing a disturbance to the electric field near the surface of the sample (see the direction of the electric field in Figure 5b). Subsequently at (*t* = 350 μs), the interaction of the sample with the electrons decreases, reshaping the electric field and restoring its intensity in the space between the LTP and the sample.

During the AAV period, ions and excited species are generated through collisions between electrons and atoms of the He-Air mixture. Figure 6b illustrates the spatiotemporal evolution of the number density of He*. Initially (*t* = 50 μs), we observed constant production of He* solely within the dielectric tube (*t* = 150–250 μs), resulting in its density remaining almost constant. Moreover, during the AAV transition from positive to negative voltages, an increase of He* by approximately 4 orders of magnitude was observed; it was particularly intense near the exit of the LTP probe. The evolution of the plasma ions was evaluated by using N_2_^+^ ions. N_2_^+^ production remained almost constant within the capillary and in the vicinity of the LTP probe. Compared to He*, N_2_^+^ density was roughly 4 orders of magnitude higher within the dielectric tube and approximately 1 order of magnitude greater in the space between the sample and the LTP output.

**Figure 6:**
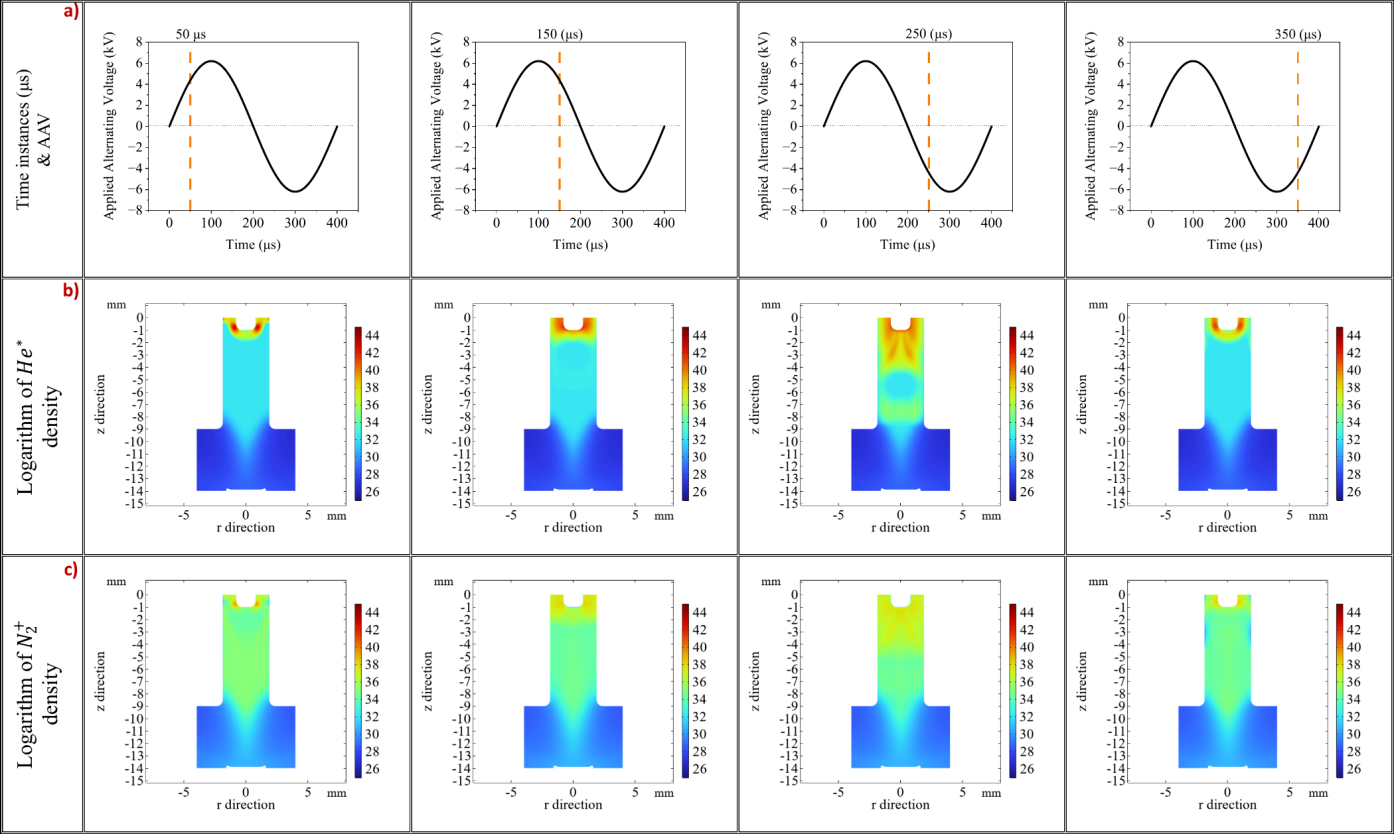
In the first line of the table noted by letter (a) is given the AAV along with four time instances of interest depicted by orange lines. In the second line of the table noted by letter (b) is given the He* metastable number density for the same time instances of the AAV. In the third line of the table noted by letter (c) is given the N_2_^+^ number density for the same time instances of the AAV. The color bar represents the natural logarithm of the number density (ln­(*n*_*e*_)). The displayed logarithmic values correspond to an absolute electron density range from approximately 2.0 × 10^11^ m^–3^ (ln­(*n*) ≈ 26) to 1.3 × 10^19^m^–3^(ln­(*n*) ≈ 44).

We anticipated that ions related to air, such as oxygen species, would be produced solely within the mixing region between the helium flow and the sample. However, the inner electrode works as a cathode in the first half of the AAV cycle and as an anode in the second half, resulting in the observed diffusion of air ions into the vicinity of the LTP probe. This phenomenon is illustrated in Figure 7b. Initially (*t* = 50–150 μs), the production and diffusion of O_2_^–^ into the interior volume of the LTP probe began, while at later times (*t* = 250–350 μs), a modest diffusion from the capillary volume to the vicinity of the LTP was also observed. Simultaneously, the positive ions O_2_^+^ are produced inside the LTP probe and diffused toward the sample. Consequently, all reactive species produced by the ionization of the Air-He mixture periodically oscillate toward and away from the sample surface, driven by the polarity of the inner electrode of the LTP probe. This periodic movement leads to chemical stress on the sample due to surface reactions likely caused by reactive plasma ions and the accumulation of charges in the surface of the sample.

**Figure 7:**
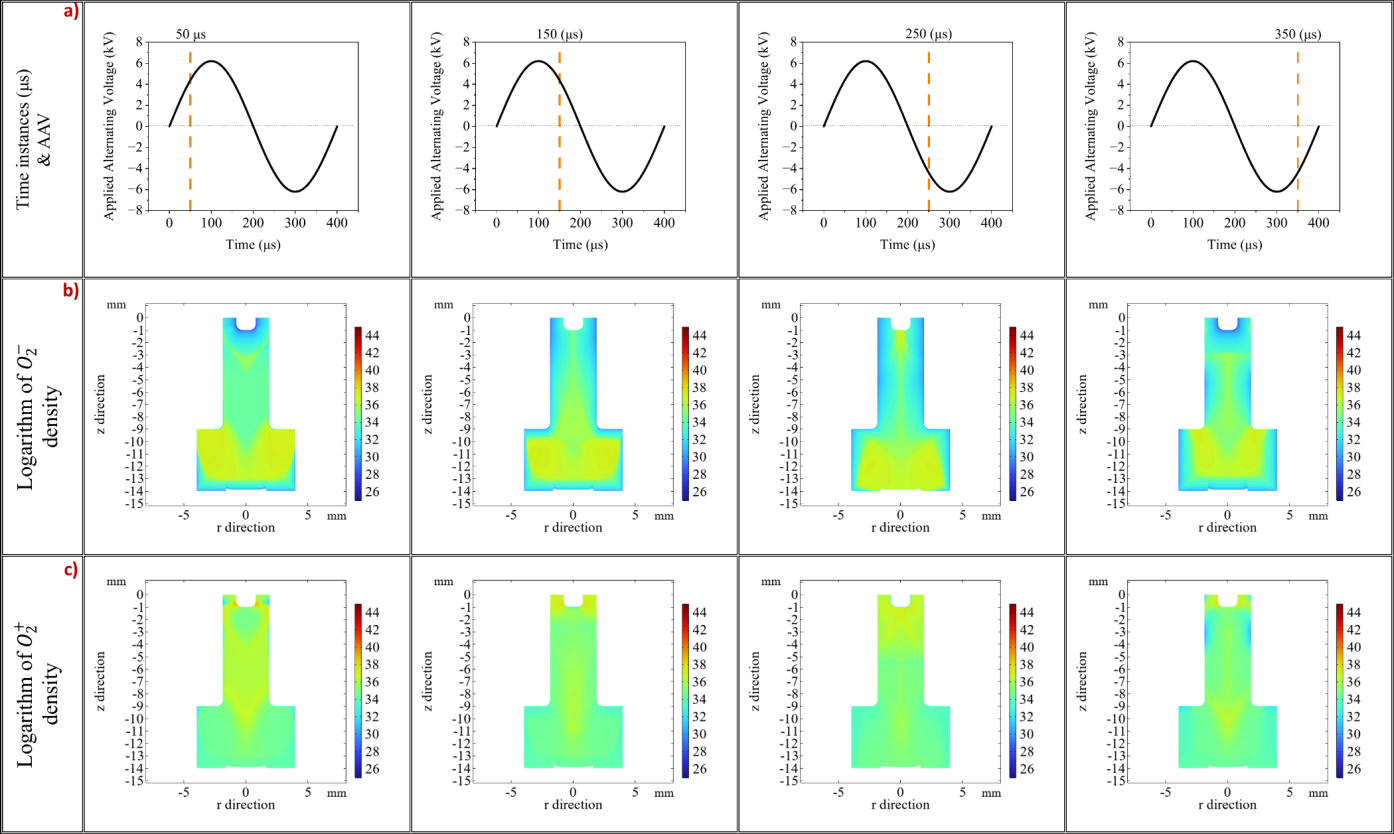
In the first line of the table noted by letter (a) is given the AAV along with four-time instances of interest depicted by orange lines. In the second line of the table noted by letter (b) is given the O_2_^–^ number density for the same time instances of the AAV. In the third line of the table noted by letter (c) is given the O_2_^+^ number density for the same time instances of the AAV. The color bar represents the natural logarithm of the number density (ln­(*n*_*e*_)). The displayed logarithmic values correspond to an absolute electron density range from approximately 2.0 × 10^11^ m^–3^ (ln­(*n*) ≈ 26) to 1.3 × 10^19^m^–3^ (ln­(*n*) ≈ 44).

### Electric Displacement of the Sample

The aforementioned analysis highlights the pivotal role played by free electrons and reactive species in the desorption mechanisms of analytes from surfaces. Notably, the dynamic distribution of electrons, He*, N_2_^+^, O_2_^–^, and O_2_^+^, in space resulted in a variable electric field and charge accumulation on the sample surface, leading to two stages that likely contribute to the desorption and ionization of the analyte from the surface.

Figure 8a,b shows the electric displacement components along with the amplitude of the electric field formed on the sample surface. In the time frame from *t* = 0 to 150 μs (positive part of the AAV cycle), the electric displacement on the sample surface increased, with the maximum value observed close to the periphery of the sample for both components. The uneven shape of the sample due to the coffee ring effect is likely inducing the differences in electric displacement. The electric field mirrors this behavior with a value around 80–100 V/mm on the surface of the sample (see Figure 8c for *t* ∼ 50 μs). During this phase, the formed electric field stresses the sample and likely disrupts the intermolecular forces formed between the molecules in the sample and the substrate/surface. Specifically, we can observe that during the positive half of the AAV cycle the strongest component on the surface of the sample, in absolute value, is in the *z* direction (Figure 8b), being the direction of desorption. Furthermore, simultaneously, it appears that the positive charges spread radially toward the periphery of the sample, while the negatives tend to align with the *z*-direction, which is perpendicular to the sample and close to the vicinity of the LTP probe. This means that the electric displacement within the sample, that is, the desorption efficiency, is higher in the negative ion mode. This result is consistent with the desorption and detection of nonvolatile nitroaromatic explosives with low vapor pressures.^19,20^

**Figure 8:**
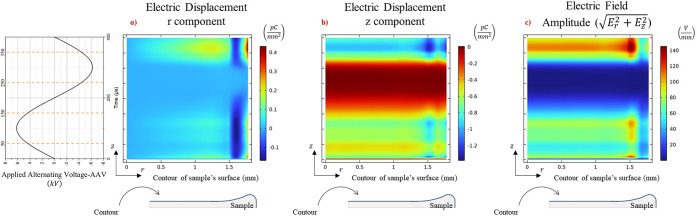
Spatiotemporal components of the electric displacement on the contour of the sample’s surface during the interaction with plasma, showing (a) the *r*-component of the electric displacement, (b) the *z*-component of the electric displacement, and (c) the amplitude of the electric field. The *x*-axis corresponds to the length of the contour of the sample, as schematically represented by the blue line in the 2D axisymmetric sketch. The *y*-axis corresponds to time for a full cycle of the AAV as depicted in the left part of the image.

In the interval from *t* = 150 μs to *t* = 300 μs, the negative surface charge of the sample is dominant but the electric field is close to zero (for most of the negative half cycle). In this phase, while there is a charge on the surface of the sample, the surface electric field returns to a relaxed state. In this interval, the *z* component of the electric displacement on the surface doubles compared to the first half of the voltage cycle (see Figure 8b for *t* ∼ 350 μs). Therefore, from the spatiotemporal profile of electric displacement and the surface electric field, it seems that the sample stressed and distressed in a periodic way. That is, large periodic forces are exerted on the bonds that bind the analyte to the glass substrate. This repetitive stress on the surface bonds probably culminates in the eventual desorption of the analyte from the substrate. Once desorbed, the analyte promptly interacts with the reactive plasma species, whose ionization mechanisms have been previously studied.

The simulation results suggest an additional component triggering desorption. Figure 9 provides a spatiotemporal representation of the mean electron energy. Notably, the maximum energy observed is approximately 2.5 eV. This energy range may overcome both substrate–analyte and analyte–analyte (condensed-phase) interactions. This proposed electron-induced desorption mechanism would complement the established thermal and laser desorption effects. Further analysis is warranted to validate this interpretation and to elucidate any associated kinetic behaviors or thresholds.

**Figure 9:**
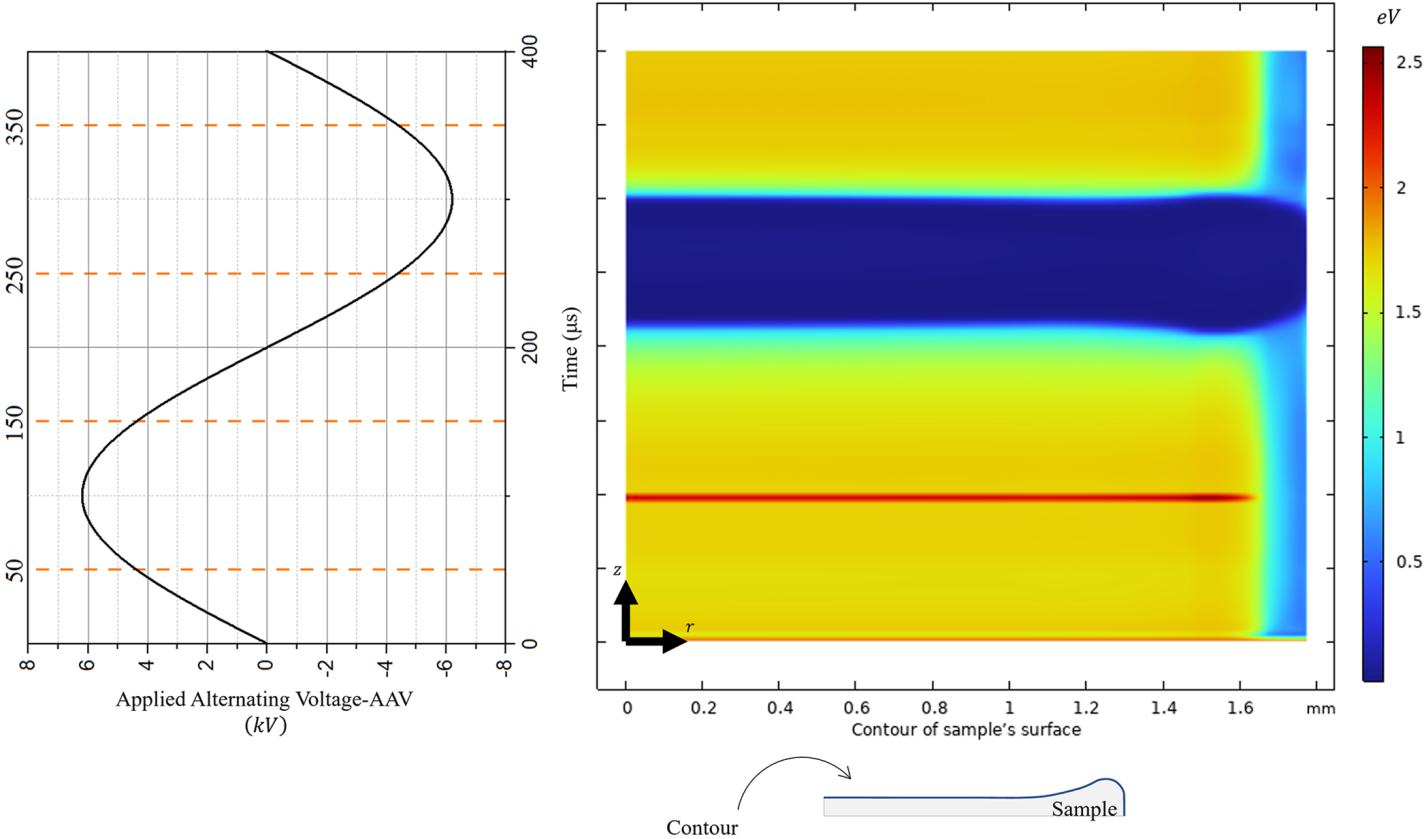
Spatiotemporal mean electron energy. The *x*-axis corresponds to the length of the contour of the sample as schematically represented by the blue line in the 2D axisymmetric sketch. The *y*-axis corresponds to the time for a cycle of the AAV.

## Conclusions

This study investigated the LTP probe plasma-based mechanisms to desorb analytes from solid samples placed on a glass substrate. The findings revealed that the electric field, in combination with the surface charge accumulated on the analyzed sample, contributed to weakening of the interactions between the analytes and the glass substrate. Furthermore, the observed periodic charge accumulation on the sample surface suggested cyclical polarization of the analyte molecules. Ultimately, this periodic polarization, combined with the weakening of surface bonds, is anticipated to result in the release of the analyte from the sample surface. Moreover, it was observed that the mean electron energy may be an extra desorption mechanism as the maximum energy is ca. 2 eV, which is close to the energy of intermolecular forces that bond the analyte to the glass substrate.

In summary, the desorption of substances facilitated by LTP appears to be a periodic process produced by reactive species within the plasma. Furthermore, this modeling framework lays the groundwork for future in silico studies aimed at optimizing LTP probe parameters (e.g., geometry and voltage), provided that the significant computational challenges associated with model scale and complexity can be addressed with adequate resources.

## Supplementary Material



## Notes

Reactions and surface reactions included in the plasma model (Tables S-1, S-2) (PDF)

## Abbreviations

AAV: applied alternating voltage
ADI-MS: ambient desorption ionization mass spectrometry
CFD: computational fluid dynamics
D/I: desorption/ionization
DBD: dielectric barrier discharge
LTP: low-temperature plasma
MS: mass spectrometry
DART: Direct Analysis in Real Time
DoF: degrees of freedom
