# Supplementary material for: Insight into Desorption Mechanisms in a Helium Low-Temperature Plasma Ionization Source Using Computational Simulations
Source: J Am Soc Mass Spectrom. 2025 Sep 12;36(10):2151–63. doi: 10.1021/jasms.5c00171 (PMC12492398; doi:10.1021/jasms.5c00171)
Supplement: Supplementary file 1 [file js5c00171_si_001.pdf]

Supporting information

Insight into Desorption Mechanisms in a Helium Low-Temperature Plasma (LTP) Ionization Source using Computational Simulations

Odhisea Gazeli*[a,b], Constantinos Lazarou[a,b], Marcos Bouza[c], David Moreno-González[c], Charalambos Anastassiou[a,b], Joachim Franzke[d], Juan F. García-Reyes*[c] and George E. Georghiou[a,b]

[a] FOSS Research Centre for Sustainable Energy, Department of Electrical and Computer Engineering, University of Cyprus, Nicosia 1678, Cyprus. [b]ENAL Electromagnetics and Novel Applications Lab, Department of Electrical and Computer Engineering, University of Cyprus, Nicosia 1678, Cyprus. [c] Analytical Chemistry Research Group, Department of Physical and Analytical Chemistry, University of Jaén, 23071 Jaén, Spain. [d]Leibniz-Institut für Analytische Wissenschaften – ISAS – e.V., Bunsen-Kirchhoff-Str. 11, 44139 Dortmund, Germany.

*Corresponding author. E-mail address: gazeli.odhisea@ucy.ac.cy, jfgreyes@ujaen.es

Contents

Table S-1. Reactions included in the Plasma Model

Table S-2. Surface Reactions included in the Plasma Model.

Table S-1: Reactions included in the Plasma Model

Reaction No.	Reaction[a]	Rate constant[b]	Energy (eV)
1	$e + He \rightarrow e + He$	$f(\varepsilon, n)$	0
2	$e + He \rightarrow e + He^{*}$	$f(\varepsilon, n)$	19.82
3	$e + He \rightarrow 2e + He^{+}$	$f(\varepsilon, n)$	24.58
4	$e + He^{*} \rightarrow e + He$	$2.9 \cdot 10^{-15}$	-19.82
5	$e + He^{+} \rightarrow He^{*}$	$6.76 \cdot 10^{-19} T_e^{-0.5}$	0
6	$e + He^{+} + He \rightarrow He^{*} + He$	$7.4 \cdot 10^{-47} T_e^{-0.5}$	0
7	$2e + He^{+} \rightarrow He^{*} + e$	$7.8 \cdot 10^{-50} \left(\frac{T_e}{T_g}\right)^{-4.4}$	-4.78
8	$e + He^{*} \rightarrow 2e + He^{+}$	$2.54 \cdot 10^{-13} T_e^{-0.1241} e^{-\frac{5.725}{T_e}}$	4.78
9	$2He^{*} \rightarrow He^{+} + He + e$	$8.7 \cdot 10^{-16} \left(\frac{T_g}{300}\right)^{0.5}$	-15.8
10	$e + He_2 \rightarrow e + 2He$	$3.8 \cdot 10^{-15}$	-17.9
11	$e + He_2 \rightarrow 2e + He_2^{+}$	$9.75 \cdot 10^{-16} T_e^{0.71} e^{-\frac{3.4}{T_e}}$	3.4
12	$2He^{*} \rightarrow He_2^{+} + e$	$2.03 \cdot 10^{-15} \left(\frac{T_g}{300}\right)^{0.5}$	-18.2
13	$He^{*} + He_2 \rightarrow He^{+} + 2He + e$	$5 \cdot 10^{-16} \left(\frac{T_g}{300}\right)^{0.5}$	-13.5
14	$He^{*} + He_2 \rightarrow He_2^{+} + He + e$	$2 \cdot 10^{-15} \left(\frac{T_g}{300}\right)^{0.5}$	-15.9
15	$2He_2 \rightarrow H_e^{+} + 3He + e$	$3 \cdot 10^{-16} \left(\frac{T_g}{300}\right)^{0.5}$	-11.3
16	$2He_2 \rightarrow He_2^{+} + 2He + e$	$1.2 \cdot 10^{-15} \left(\frac{T_g}{300}\right)^{0.5}$	-13.7
17	$e + N_2 \rightarrow N_2^{+} + 2e$	$f(\varepsilon, n)$	15.5
18	$e + O_2 \rightarrow O(1s) + O + e$	$f(\varepsilon, n)$	9.97
19	$e + O_2 \rightarrow O + O^{-}$	$f(\varepsilon, n)$	0
20	$e + O_4 \rightarrow 2O_2$	$2.25 \cdot 10^{-13} T_e^{-0.5}$	0
21	$e + O_2 + He \rightarrow He + O_2^{-}$	$1 \cdot 10^{-43}$	0
22	$e + O_2 \rightarrow O_2^{+} + 2e$	$f(\varepsilon, n)$	12.1
23	$e + O_2 \rightarrow e + 2O$	$f(\varepsilon, n)$	6
24	$e + O_2 \rightarrow e + O(1d) + O^{-}$	$f(\varepsilon, n)$	8.4

25	$e + O_2 \to e + O_2(A)$	$f(\varepsilon, n)$	0.977
26	$e + O_2 \to O + O_2(B)$	$f(\varepsilon, n)$	1.627
27	$e + O_2 \to O + O$	$1.2 \cdot 10^{-14} T_e^{-0.7}$	0
28	$2e + O_2^+ \to O_2 + e$	$7.18 \cdot 10^{-39} T_e^{-4.5}$	0
29	$e + O_2^+ + O^2 \to 2O_2$	$2.49 \cdot 10^{-41} T_e^{-1.5}$	0
30	$e + N_2 \to e + N_2(A^3 \sum_u^+ (v = 0, \dots, 4))$	$f(\varepsilon, n)$	6.2
31	$e + N_2 \to e + N_2(A^3 \sum_u^+ (v = 5, \dots, 9))$	$f(\varepsilon, n)$	7
32	$e + N_2 \to e + N_2(A^3 \sum_u^+ (v > 9))$	$f(\varepsilon, n)$	7.8
33	$e + N_2 \to e + N_2(B^3\Pi_g)$	$f(\varepsilon, n)$	7.4
34	$e + N_2 \to e + N_2(W^3\Delta_u)$	$f(\varepsilon, n)$	7.4
35	$e + N_2 \to e + N_2(B^3\Sigma_u^-)$	$f(\varepsilon, n)$	8.2
36	$e + N_2 \to e + N_2(a^1\Sigma_u^-)$	$f(\varepsilon, n)$	8.4
37	$e + N_2 \to e + N_2(a^1\Pi_g)$	$f(\varepsilon, n)$	8.6
38	$e + N_2 \to e + N_2(W^1\Delta_u)$	$f(\varepsilon, n)$	8.9
39	$e + N_2 \to e + N_2(C^3\Pi_u)$	$f(\varepsilon, n)$	11
40	$e + N_2 \to e + N_2(E^3\Sigma_g^+)$	$f(\varepsilon, n)$	11.9
41	$e + N_2 \to e + N_2(a^1\Sigma_g^+)$	$f(\varepsilon, n)$	12.3
42	$e + O_2 \to e + O_2(v = 1)$	$f(\varepsilon, n)$	0.19
43	$e + O_2 \to e + O_2(v = 2)$	$f(\varepsilon, n)$	0.38
44	$e + O_2 \to e + O_2(v = 3)$	$f(\varepsilon, n)$	0.6
45	$e + O_2 \to e + O_2(v = 4)$	$f(\varepsilon, n)$	0.8
46	$e + 2O_2 \to O_2 + O_2^-$	$2.26 \cdot 10^{-42}$	0
47	$e + N_2 \to e + N_2$	$f(\varepsilon, n)$	0
48	$e + N_2 \to e + N_2\ (v = 1)$	$f(\varepsilon, n)$	0.3
49	$e + N_2 \to e + N_2\ (v = 2)$	$f(\varepsilon, n)$	0.6
50	$e + N_2 \to e + N_2\ (v = 3)$	$f(\varepsilon, n)$	0.9
51	$e + N_2 \to e + N_2\ (v = 4)$	$f(\varepsilon, n)$	1.1
52	$e + O_2 \to e + O_2$	$f(\varepsilon, n)$	0

53	$e + He \rightarrow e + He(3s^3S)$	$f(\varepsilon, n)$	22.718
54	$He^* + 2He \rightarrow 3He$	$2 \cdot 10^{-46}$	0
55	$e + He_2 \rightarrow He^* + He$	$7.12 \cdot 10^{-21} \left(\frac{T_g}{300}\right)^{1.5}$	0
56	$e + He_2 + He \rightarrow He^* + 2He$	$3.5 \cdot 10^{-39}$	0
57	$e + He_2^+ + He \rightarrow He_2 + He$	$1.5 \cdot 10^{-39}$	0
58	$e + He_2^+ \rightarrow 2He$	10^{-14}	0
59	$e + He_2^+ + He \rightarrow 3He$	$2 \cdot 10^{-39}$	0
60	$2e + He_2^+ \rightarrow He^* + He + e$	$2.8 \cdot 10^{-32}$	0
61	$2e + He_2^+ \rightarrow He_2 + e$	$1.2 \cdot 10^{-33}$	0
62	$He^* + 2He \rightarrow He_2 + He$	$2 \cdot 10^{-46}$	0
63	$He^+ + 2He \rightarrow He_2^+ + He$	$1.4 \cdot 10^{-43} \left(\frac{T_g}{300}\right)^{-0.6}$	0
64	$He_2 + He \rightarrow 3He$	$1.5 \cdot 10^{-21}$	0
65	$e + N_2^+ \rightarrow 2N$	$2.540 \cdot 10^{-12} (T_e)^{-0.5}$	0
66	$e + N_4^+ \rightarrow 2N_2$	$3 \cdot 10^{-13}$	0
67	$e + N_2^+ \rightarrow N_2 + e$	$3.17 \cdot 10^{-42}$	0
68	$e + N_4^+ \rightarrow 2N_2 + e$	$3.17 \cdot 10^{-42}$	0
69	$N_2^+ + N_2 + H_e \rightarrow N_4^+ + He$	$8.9 \cdot 10^{-42} \left(\frac{T_g}{300}\right)^{-1.54}$	0
70	$He^+ + N_2 \rightarrow He + N_2^+$	$6.15 \cdot 10^{-16}$	0
71	$He_2^+ + N_2 \rightarrow 2He + N_2^+$	$1.1 \cdot 10^{-15}$	0
72	$He^+ + N_2 + He \rightarrow 2He + N_2^+$	$1.1 \cdot 10^{-41}$	0
73	$He^+ + N_2 + He \rightarrow 3He + N_2^+$	$1.6 \cdot 10^{-41}$	0
74	$He^* + N_2 \rightarrow N_2^+ + He + e$	$5 \cdot 10^{-17}$	0
75	$He_2^+ + N_2 \rightarrow N_2^+ + 2He + e$	$5 \cdot 10^{-17}$	0
76	$O_2^+ + O^- + He \rightarrow O_2 + O + He$	$2 \cdot 10^{-37} \left(\frac{T_g}{300}\right)^{-2.5}$	0
77	$O_2^+ + O^- + O_2 \rightarrow O_2 + O + O_2$	$2 \cdot 10^{-37} \left(\frac{T_g}{300}\right)^{-2.5}$	0
78	$O_2^+ + O_2^- + He \rightarrow 2O_2 + He$	$2 \cdot 10^{-37} \left(\frac{T_g}{300}\right)^{-2.5}$	0
79	$O_2^+ + O_2^- + O_2 \rightarrow 3O_2$	$2 \cdot 10^{-37} \left(\frac{T_g}{300}\right)^{-2.5}$	0

80	$O_4^+ + O^- + He \rightarrow 2O_2 + O + He$	$2 \cdot 10^{-37} \left(\frac{T_g}{300}\right)^{-2.5}$	0
81	$O_4^+ + O^- + O_2 \rightarrow 2O_2 + O + O_2$	$2 \cdot 10^{-37} \left(\frac{T_g}{300}\right)^{-2.5}$	0
82	$O_4^+ + O^- + Oe \rightarrow 2O_2 + O + O_2$	$2 \cdot 10^{-37} \left(\frac{T_g}{300}\right)^{-2.5}$	0
83	$O_4^+ + O_2^- + He \rightarrow 3O_2 + He$	$2 \cdot 10^{-37} \left(\frac{T_g}{300}\right)^{-2.5}$	0
84	$O_4^+ + O_2^- + O_2 \rightarrow 3O_2 + O_2$	$5.8 \cdot 10^{-43} \left(\frac{T_g}{300}\right)^{-3.1}$	0
85	$O_2^+ + O_2 + He \rightarrow O_4^+ + He$	$3.3 \cdot 10^{-12} e^{-\frac{5030}{T_g}} \left(\frac{T_g}{300}\right)^{-4}$	0
86	$O_4^+ + O_2 \rightarrow O_2^+ + 2O_2$	$2.54 \cdot 10^{-16}$	0
87	$He^* + O_2 \rightarrow O_2^+ + He + e$	$10^{-16} e^{-\frac{5030}{T_g}} \left(\frac{T_g}{300}\right)^{0.5}$	0
88	$He_2 + O_2 \rightarrow O_2^+ + 2He + e$	$1.5 \cdot 10^{-21}$	0
89	$He_2 + O_2 \rightarrow 2He + O_2$	$10^{-15} \left(\frac{T_g}{300}\right)^{0.5}$	0
90	$He_2^+ + O_2 \rightarrow O_2^+ + 2He$	$1.04^{-15} T_g^{-0.5}$	0
91	$N_2^+ + O_2 \rightarrow N_2 + O_2^+$	$2.5 \cdot 10^{-16}$	0
92	$N_4^+ + O_2 \rightarrow 2N_2 + O_2^+$	$2.5 \cdot 10^{-37} \left(\frac{T_g}{300}\right)^{-2.5}$	0
93	$N_2^+ + O^- + O_2 \rightarrow O + O_2 + N_2$	$2 \cdot 10^{-37} \left(\frac{T_g}{300}\right)^{-2.5}$	0
94	$N_2^+ + O^- + N_2 \rightarrow O + 2N_2$	$2 \cdot 10^{-37} \left(\frac{T_g}{300}\right)^{-2.5}$	0
95	$N_2^+ + O_2^- + O_2 \rightarrow 2O_2 + N_2$	$2 \cdot 10^{-37} \left(\frac{T_g}{300}\right)^{-2.5}$	0
96	$O_4^+ + O_2^- + N_2 \rightarrow 3O_2 + N_2$	$10^{-37} \left(\frac{T_g}{300}\right)^{-2.5}$	0
97	$O_4^+ + O^- + N_2 \rightarrow 2O_2 + O + N_2$	$10^{-37} \left(\frac{T_g}{300}\right)^{-2.5}$	0
98	$O_2^+ + O^- + N_2 \rightarrow O_3 + N_2$	$2 \cdot 10^{-37} \left(\frac{T_g}{300}\right)^{-2.5}$	0
99	$N_4^+ + O^2 \rightarrow O_2 + N_2 + N_2^+$	$2.5 \cdot 10^{-16}$	0
100	$O_4^+ + N_2 \rightarrow O_2 + N_2 + O_2^+$	$10^{-11} e^{-\frac{5400}{T_g}}$	0

101	$e + O_2 + N_2 \rightarrow N_2 + O_2^-$	$1.24 \cdot 10^{-43}\left(\frac{T_g}{300}\right)^{-0.5}$	0
102	$He^* + N_2 + He \rightarrow N_2^+ + 2He + e$	$3.3 \cdot 10^{-42}$	0
103	$O_2^+ + O_2 + O_2 \rightarrow O_4^+ + O_2$	$2.4 \cdot 10^{-42}\left(\frac{T_g}{300}\right)^{3.2}$	0
104	$He_2 + N_2 \rightarrow 2He + N_2$	$1.5 \cdot 10^{-21}$	0
105	$O_2^+ + O^- + N_2 \rightarrow 2He + N_2$	$2 \cdot 10^{-37}\left(\frac{T_g}{300}\right)^{-2.5}$	0
106	$O_2^+ + O^- + N_2 \rightarrow O_2 + O + N_2$	$2 \cdot 10^{-37}\left(\frac{T_g}{300}\right)^{-2.5}$	0
107	$O_2^+ + O_2^- + N_2 \rightarrow 2O_2 + N_2$	$6 \cdot 10^{-39}\left(\frac{T_e}{T_g}\right)^{-1.5}$	0
108	$N_2^+ + O_2 + e \rightarrow O_2 + N_2$	$6 \cdot 10^{-39}\left(\frac{T_e}{T_g}\right)^{-1.5}$	0
109	$O_2^+ + N_2 + e \rightarrow O_2 + N_2$	$6 \cdot 10^{-39}\left(\frac{T_e}{T_g}\right)^{-1.5}$	0
110	$He + He^* + O_2 \rightarrow O_2^+ + 2He + e$	$1.6 \cdot 10^{-43}$	0
111	$O_2^+ + O^- + O_2 \rightarrow O_2 + O_3$	$2 \cdot 10^{-37}\left(\frac{T_g}{300}\right)^{-2.5}$	0
112	$N_2^+ + 2N_2 \rightarrow N_4^+ + N_2$	$5 \cdot 10^{-41}$	0
113	$e + He^* \rightarrow He(2P^3P) + hf$	1.547	0

a) He^* *represents* $He(2^3S)$ *and* $He(2^1S)$*;*

b) Rate coefficients have units of s^{-1}*,* m^3s^{-1}*,* m^6s^{-1} *for one, two and three body reactions, respectively;* Te *has units* eV*;* T_g *has units of* K*.* $f(\varepsilon, n)$ *indicates the rate coefficient as a function of the mean electron energy calculated from the solution of the Boltzmann equation and the air mole fraction.*

Table S-2. Surface Reactions included in the Plasma Model.

Surface Reaction No.	Reaction	Secondary emission coefficients	Mean energy	Boundary
1	$He^* \rightarrow He$	0	0	1-2-3-4-5 & 6-7
2	$He^+ \rightarrow He$	0.1	5	1-2-3-4-5 & 6-7
3	$He_2 \rightarrow 2He$	0	0	1-2-3-4-5 & 6-7
4	$He_2^+ \rightarrow 2He$	0.1	5	1-2-3-4-5 & 6-7
5	$N_2^+ \rightarrow N_2$	0.1	5	1-2-3-4-5 & 6-7
6	$N_4^+ \rightarrow 2N_2$	0.1	3	1-2-3-4-5 & 6-7
7	$O^- \rightarrow 0.5O_2$	0	0	1-2-3-4-5 & 6-7
8	$O_4^+ \rightarrow 2O_2$	0.1	3	1-2-3-4-5 & 6-7
9	$O_2^- \rightarrow O_2$	0	0	1-2-3-4-5 & 6-7
10	$O_2^+ \rightarrow O_2$	0.1	3	1-2-3-4-5 & 6-7
11	$He(2P^3P) \rightarrow He$	0	0	1-2-3-4-5 & 6-7
12	$He^* \rightarrow He$	0	0	5-6
13	$He^+ \rightarrow He$	0	0	5-6
14	$He_2 \rightarrow 2He$	0	0	5-6
15	$He_2^+ \rightarrow 2He$	0	0	5-6
16	$N_2^+ \rightarrow N_2$	0	0	5-6
17	$N_4^+ \rightarrow 2N_2$	0	0	5-6
18	$O_4^+ \rightarrow 2O_2$	0	0	5-6
19	$O_2^+ \rightarrow O_2$	0	0	5-6
20	$O^- \rightarrow 0.5O_2$	0	0	5-6
21	$O_2^- \rightarrow O_2$	0	0	5-6
22	$He(2P^3P) \rightarrow He$	0	0	5-6
